# Mechanistic differences between phenotypes of chronic lung allograft dysfunction after lung transplantation

**DOI:** 10.1111/tri.12341

**Published:** 2014-06-17

**Authors:** Monika I Suwara, Bart M Vanaudenaerde, Stijn E Verleden, Robin Vos, Nicola J Green, Chris Ward, Lee A Borthwick, Elly Vandermeulen, Jim Lordan, Dirk E Van Raemdonck, Paul A Corris, Geert M Verleden, Andrew J Fisher

**Affiliations:** 1 Fibrosis Research Group, Institute of Cellular Medicine, Newcastle UniversityNewcastle upon Tyne, UK; 2 Department of Clinical and Experimental Medicine, Lab of Pneumology, Leuven Lung Transplantation Unit, Katholieke Universiteit Leuven and University Hospital GasthuisbergLeuven, Belgium; 3 Institute of Transplantation, Freeman HospitalNewcastle Upon Tyne, UK

**Keywords:** epithelial cell damage, inflammation, lung transplantation, neutrophil, phenotyping, rejection

## Abstract

Distinct phenotypes of chronic lung allograft dysfunction (CLAD) after lung transplantation are emerging with lymphocytic bronchiolitis (LB)/azithromycin reversible allograft dysfunction (ARAD), classical or fibrotic bronchiolitis obliterans syndrome (BOS), and restrictive allograft syndrome (RAS) proposed as separate entities. We have additionally identified lung transplant recipients with prior LB, demonstrating persistent airway neutrophilia (PAN) despite azithromycin treatment. The aim of this study was to evaluate differences in the airway microenvironment in different phenotypes of CLAD. Bronchoalveolar lavage (BAL) from recipients identified as stable (control), LB/ARAD, PAN, BOS, and RAS were evaluated for differential cell counts and concentrations of IL-1α, IL-1β, IL-6, IL-8, and TNF-α. Primary human bronchial epithelial cells were exposed to BAL supernatants from different phenotypes and their viability measured. BOS and RAS showed increased BAL neutrophilia but no change in cytokine concentrations compared with prediagnosis. In both LB/ARAD and PAN, significant increases in IL-1α, IL-1β, and IL-8 were present. BAL IL-6 and TNF-α concentrations were increased in PAN and only this phenotype demonstrated decreased epithelial cell viability after exposure to BAL fluid. This study demonstrates clear differences in the airway microenvironment between different CLAD phenotypes. Systematic phenotyping of CLAD may help the development of more personalized approaches to treatment.

## Introduction

Lung transplantation (LTx) is accepted as a well-established therapeutic option for patients with end-stage lung disease, yet long-term survival remains less than that achieved in transplantation of other solid organs, with a median survival of 5 years 1. Predominantly, this is limited by the development of chronic rejection, which affects 45–50% of LTx patients within 5 years and accounts for more than 30% of late mortality 2. Chronic rejection was defined as an abnormal, airway-centered, inflammatory response resulting in fibroproliferative small airway obliteration. The cellular mechanisms driving chronic rejection remain poorly elucidated, but current consensus suggests that both alloantigen-dependent 3,4 and alloantigen-independent insults to airway epithelium lead to an influx of inflammatory cells, chronic neutrophilic inflammation, deregulated repair, and resultant fibrotic plugging of the small airways presenting in the typical obliterative bronchiolitis (OB) lesions 5,6. A multitude of risk factors have been identified, which include HLA mismatch 7, acute rejection episodes 8, recipient age 9, CMV 10, community respiratory viral infections 11, pseudomonal colonization 12, lymphocytic bronchitis/bronchiolitis 13, gastroesophageal reflux 14, air pollution 15, and ischemia–reperfusion injury 16. Additionally, recent observations suggest that proinflammatory cytokines such as IL-1 family members, TNF-α 17, and IL-17 18,19 may be the key mediators driving these insults, via subsequent induction of the potent neutrophilic chemoattractant protein IL-8. One of the characteristics of chronic rejection is damage to the bronchial epithelium 20. Previous studies have demonstrated that damage to epithelium may result in the release of alarmins, including IL-1α, which in a paracrine manner may trigger expression of proinflammatory cytokines in lung fibroblasts and other epithelial cells 21.

Clinically, chronic rejection manifests with persistent cough, increased sputum production, dyspnea, and coarse crackles and can be quantified functionally by a progressive loss of forced expiratory volume in one second (FEV_1_). Traditionally, this clinical presentation would have been labeled as bronchiolitis obliterans syndrome (BOS) but more recently has been termed chronic lung allograft dysfunction (CLAD) 22,23. CLAD is used to reflect the heterogeneous nature of the pathophysiology characterized by different levels of airway neutrophilia, airway and parenchymal fibrosis, distinct histological features, and differing responsiveness to macrolide antibiotics 23. On the basis of these criteria, it has recently been proposed that CLAD can be differentiated into two subphenotypes: classical BOS and restrictive allograft syndrome (RAS) 24. Azithromycin reversible airways dysfunction (ARAD), a clinical condition characterized by an obstructive decline in pulmonary function, increased BAL neutrophils and improvement in both these findings with azithromycin treatment, is currently considered as a separate entity, which due to its reversible nature cannot be classified as CLAD anymore 24. The only very recently identified CLAD phenotype, RAS, is characterized by parenchymal infiltrates on HRCT scan histological appearance of parenchymal fibrosis usually in combination with the typical OB lesions; and a restrictive pulmonary function defect 25. Classical BOS, on the other hand shows no neutrophilic airway inflammation, is not reversible by azithromycin and has radiological evidence of air trapping due to OB causing airway obliteration 26. ARAD associated with a neutrophilic airway inflammation that can be attenuated by azithromycin therapy is mechanistically identical to lymphocytic bronchiolitis (LB) (also called B grade acute rejection) given that both are IL-17-mediated conditions. Currently, in both the Leuven and Newcastle lung transplant centers, we are observing lung transplant patients, who develop persistent, neutrophilic airway inflammation despite azithromycin therapy for more than 3-week duration (denominated PAN or persistent airway neutrophilia in the current paper) in the absence of submucosal demonstrable IL-17 T lymphocytes 27.

The aim of this study was not to validate the new CLAD terminology 27, but to investigate any mechanistic differences between these clinical subphenotypes, which may help us direct future basic and clinical research toward a personalized treatment to further improve outcome after LTx. The mechanistic elements being evaluated are airway inflammation, inflammatory cytokine concentrations, IL-1α alarmin concentrations, and epithelial cell viability.

## Material and methods

### Patients

**Figure 1:**
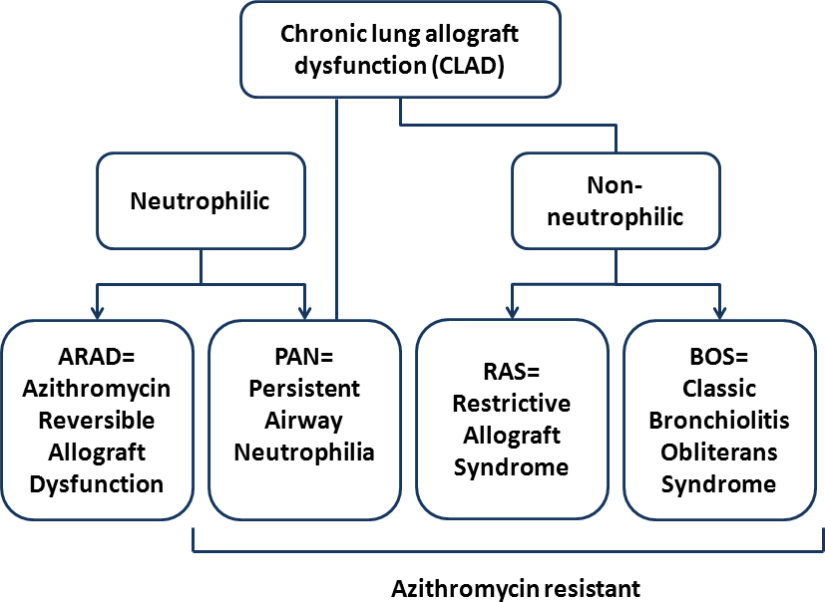
Proposed dichotomy of chronic allograft dysfunction.

Since October 2001, all lung transplant recipients at the Leuven University Hospital have been enrolled in a routine prospective bronchoscopy study. Physical and radiological examination, systemic C-reactive protein (CRP), lung function, and bronchoscopy with bronchoalveolar lavage (BAL) collection are performed at fixed time points after LTx or in case of suspected complication. Bronchoalveolar lavage samples were obtained from lung transplant recipients from the Leuven Lung Transplant programme with hospital ethical committee approval (s51577), and all participants provided written informed consent. Bronchoalveolar lavage procedure was performed as previously reported 24. Briefly for BAL, two aliquots of sterile saline (50 ml) were instilled in the right middle lobe or lingula. The returned fractions were pooled and processed for cell counting and supernatant collection for protein measurement and BEC stimulation. To be included in the study, the patients needed a bronchoscopy and BAL collection at the time of diagnosis of BOS, RAS, ARAD/LB, or PAN without interfering factors such as infection, post-transplant lymphoproliferative disorder (PTLD), and anastomotic complications. RAS (*n* = 9) was defined as a combination of a persistent parenchymal infiltrates on CAT scan together with a persistent TLC decline of more than 10% or a decline in FEV_1_ of more than 20% and a simultaneous decline in forced vital capacity (FVC), so that the FEV_1_/FVC ratio was 0.7 or more, compatible with a restrictive pulmonary function defect as previously reported 26,29. BOS patients (*n* = 13) were defined by a persistent decline in FEV_1_ of more than 20%, without any restrictive pattern of pulmonary function defect and without another identifiable cause or confounding factor being present. ARAD/LB patients (*n* = 10) histologically demonstrated lymphocytic inflammation around the airways (B grade acute rejection) without inflammation around the blood vessels (A grade acute rejection) and were not on azithromycin treatment for at least 3 months. PAN patients (*n* = 10) were diagnosed with prior B grade rejection and therefore were actively treated with azithromycin for at least 2 months. In this patient group, azithromycin treatment was started because of a previous episode of high BAL neutrophilia or re-transplantation (routine immunosuppression for re-transplant patients in our center includes azithromycin treatment at discharge from the hospital). No other interfering factors such as infection, post-transplant lymphoproliferative disorder (PTLD), and anastomotic complications were present. A control group was selected and matched according to the mean sample time of the whole rejection group. Additionally, as controls for BOS and RAS, samples obtained from these two groups of patients prior to diagnosis (pre-BOS and pre-RAS, respectively) were also included in the study. A proposed phenotypic dichotomy of chronic lung allograft dysfunction is presented in Fig.1. Patient characteristics are shown in Table1. Primary bronchial epithelial cells (PBEC) were obtained from a stable lung transplant recipient undergoing surveillance bronchoscopy at the Freeman Hospital, Newcastle. (The study was approved by the regional ethics committee, and the patient provided written informed consent to participate Ref. 2001/179.) IL-1α, IL-1β, IL-6, IL-8, and TNF-α proteins were measured in undiluted BAL using MSD electrochemiluminescence assay (MesoScale Discovery, Gaithersburg, US) according to the manufacturer's instructions. Protein limits of quantification were 0.63 pg/ml (IL-1α), 0.65 pg/ml (IL-1β), 0.64 pg/ml (IL-6), 3.33 pg/ml (IL-8), and 0.66 pg/ml (TNF-α).

**Table 1:** Patient characteristics.

	Control	LB/ARAD	PAN	BOS		RAS		*P*-value
Pre				Diagnosis	Pre	Diagnosis		
	*n* = 13	*n* = 10	*n* = 10	*n* = 11	*n* = 13	*n* = 9		
Median age at Tx, years	57 (55–62)	55 (44–60)	33 (22–57)	51 (29–57)		42 (31–54)		**0.010**
Sex								
Male	7	3	3	7		4		NS
Female	6	7	7	6		5		
Pre-LTx diagnosis								
Emphysema	9	7	3	4		1		**<0.0001**
Pulm. hypertension	2	0	1	0		2		
Cystic fibrosis	1	2	2	4		0		
Interstitial lung disease	0	1	0	4		3		
OB	0	0	2	1		0		
Others	1	0	2	0		3		
Type of LTx								
Single	2	3	0	1		1		NS
Double	11	7	10	12		7		
Heart–lung	0	0	0	0		1		
Blood type								
O	8	5	7	7		7		NS
A	3	3	3	3		1		
B	2	2	0	3		1		
Mean cold ischemia								
Minutes	310 (259–329)	293 (217–356)	342 (325–385)	317 (298–418)		331 (281–341)		NS
CMV match								
Yes	8	6	3	8		3		NS
No	3	3	5	4		4		
Unknown	2	1	2	1		2		
Immunosuppressives								
Steroids	13	10	9	11	12	8	7	NS
FK/cyclosporin	12/2	9/1	10/1	10/2	12/1	9/0	8/0	NS
AZA/MMF	4/2	6/2	2/2	7/1	7/2	5/2	5/1	NS
Azithromycin	1	0	10	1	1	1	0	NS
FEV_1_ (% pred)	102 (80–108)	60 (51–81)	52 (41–73)	81 (69–89)	65 (57–73)	68 (62–78)	58 (47–64)	/
Time of BAL								
Days post-Tx	731 (727–746)	584 (72–1743)	824 (351–2617)	379 (90–544)	1045 (335–2302)	735 (371–872)	916 (719–1455)	NS
BAL return Ml	44 (38–51)	40 (36–42)	34 (28–37)	48 (42–54)	50 (39–53)	37 (31–41)	37 (30–55)	**0.0082**

Results are expressed as median (IQR). LB/ARAD, lymphocytic bronchiolitis/azithromycin reversible allograft dysfunction; PAN, persistent airway neutrophilic despite azithromycin; BOS, classical bronchiolitis obliterans syndrome; RAS, restrictive allograft dysfunction; LTx, lung transplantation; CMV, cytomegalovirus; PH, pulmonary hypertension; OB, obliterative bronchiolitis, FK, tacrolimus; AZA, azathioprine; MMF, mycophenolate mofetil. One-way anova and Fisher's exact test are used; *P* < 0.05 is considered significant and significant values are shown in bold.

### Epithelial cell culture and cell viability assay

Bronchoalveolar lavage samples used to stimulate PBEC were centrifuged, and only the supernatants are used in the BEC stimulation. Human PBEC were obtained by bronchial brushings from an anonymous stable post-transplant patient and cultured on 0.5% PureCol-coated dishes in SABM (Lonza) medium. PBEC viability was performed using XTT-based toxicity assay (Abcam) according to the manufacturer's instructions. Briefly, PBEC were seeded at 2 × 10^5^ cells/ml in the final volume of 100 μl/well in a 96-well culture plate. After 24 h, medium was replaced with 1:1 BAL supernatant/SABM. Following 24-h incubation at 37°C, 10 μl of XTT reagent was added to each well. After 1 h, OD corresponding to the relative cell metabolic activity was quantified using a multiwell plate reader. Relative cell viability presented as optical density (OD) corresponds to the formation of formazan dye by dehydrogenase enzymes in metabolically active cells. All measurements were taken at the wavelength of 450 nm and normalized to blank (medium without cells with 10 μl XTT).

### Statistical analysis

Results are expressed as mean ± SEM. Kruskal–Wallis test, Mann–Whitney *U* test, Spearman's rank test, and paired Student's *t*-test were used where appropriate by Prism 4.1 software (San Diego, CA, USA).

## Results

### Patient characteristics

No significant differences were found between the patient groups regarding gender, type of transplantation, blood type, duration of cold ischemia, CMV match/mismatch, immunosuppressive treatment, CRP, or post-LTx time of BAL collection. A significant difference in age (*P* = 0.01) was present with PAN patients being younger than control patients [33 (22–57) vs. 57 (55–62) years, *P* < 0.05]. A significant difference was found in underlying disease (*P* < 0.0001). The Fisher exact test revealed that among RAS patients, significantly less emphysema and CF were present. Additionally, in classical BOS, significantly less OB and other diseases were present. The PAN group included more OB and the control group less interstitial lung disease (Table1).

### Systemic CRP and BAL cellularity

**Figure 2:**
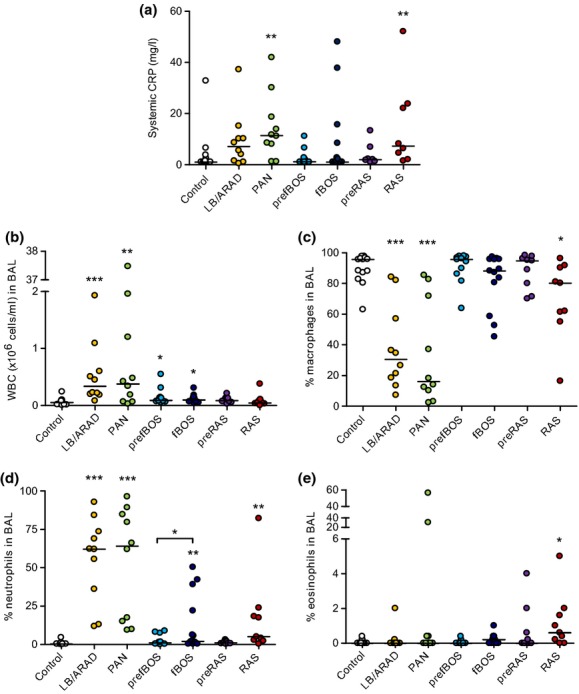
Systemic C-reactive protein and BAL cellular patterns within rejection phenotypes. The levels of systemic C-reactive protein (CRP) in blood were measured by ELISA. (a), The number of white blood cells (WBC) (b), and the percentage of macrophages (c), neutrophils (d) and eosinophils (e) in BAL of lung transplant recipients was measured by differential cell count. The different stars refer to the level of statistical significance (*P < 0.05, **P < 0.01, ***P <* *0.001).

Kruskal–Wallis anova showed significant differences for systemic CRP (*P* = 0.0075), number of BAL leukocytes (*P* = 0.0001), percentage of BAL macrophages (*P* < 0.0001), percentage of BAL neutrophils (*P* < 0.0001), and percentage of BAL eosinophils (*P* = 0.028) as shown in Fig.2(a–e). No difference was observed for the percentage of BAL lymphocytes (*P* = 0.49).

Leukocytes were increased in LB/ARAD (335 × 10^3^ (79–1925 × 10^3^) cells/ml; *P* = 0.0005), PAN (377 × 10^3^ (26–37470 × 10^3^) cells/ml; *P* = 0.0048), and BOS (94 × 10^3^ (80–306 × 10^3^) cells/ml; *P* = 0.021) compared with the control (51 × 10^3^ (5–239 × 10^3^) cells/ml). Leukocytes were also increased in pre-BOS (88 × 10^3^ (51–544 × 10^3^) cells/ml; *P* = 0.022) versus control, but not versus BOS (0.3789) as assessed by paired Student's *t*-test. BAL neutrophilia (%) was increased in the LB/ARAD [62.1% (11.8–92.8%); *P* < 0.0001], PAN [64% (9.4–96.4%); *P* < 0.0001], BOS [2% (0.4–50.4%); *P* = 0.0051], and RAS group [5% (0.4–82.2%); *P *= 0.0019] compared with the control group [0.4% (0–4.5%)]. Neutrophilia of both pre-BOS and pre-RAS groups was not different compared with the control group. Eosinophil percentage was only increased in RAS [0.6% (0–5%); *P* = 0.011] compared with the control [0% (0–0.4%)]. Serum CRP was higher in PAN [11.45 mg/l (1.2–41.9 mg/l); *P* = 0.0048] and RAS [7.25 mg/l (1.5–52.1 mg/l); *P* = 0.0091] groups compared with the control group [1 mg/ml (1–32.8 mg/ml)] (Fig.2).

### Protein profiles of cytokines in BAL

**Figure 3:**
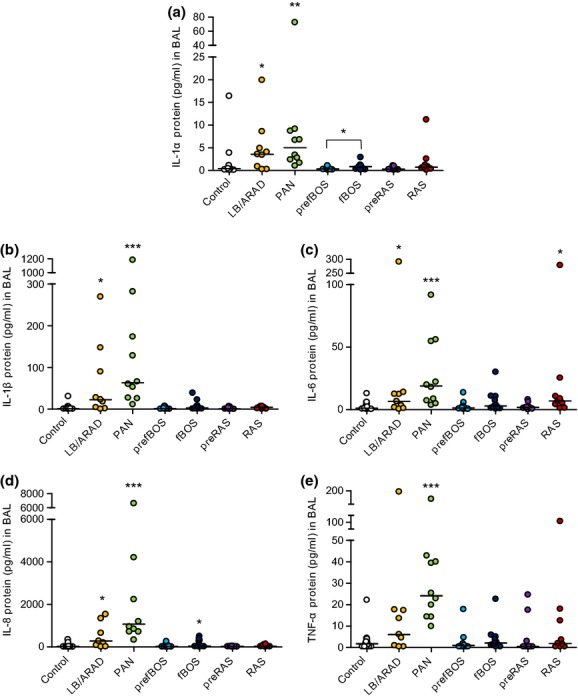
Cytokine and alarmin concentrations in BAL of lung transplant patients with different rejection phenotypes. IL-1α (a), IL-1β (b), IL-6 (c), IL-8 (d), and TNF-α (e) were measured in BAL using MSD assay. The different stars refer to the level of statistical significance (*P < 0.05, **P < 0.01, ***P < 0.001).

Kruskal–Wallis anova showed significant differences in BAL for IL-1α (*P* = 0.0002), IL-1β (*P* = 0.0001), IL-6 (*P* = 0.0006), IL-8 (*P* < 0.0001), and TNF-α (*P* < 0.0005) (Fig.3a–e). The IL-1α alarmin was significantly higher in LB/ARAD [3.54 pg/ml (0.23–19.83 pg/ml); *P* = 0.033] and PAN [5.03 pg/ml (0.99–72.6 pg/ml); *P* = 0.0011] versus control [0.41 pg/ml (0.06–16.38 pg/ml)]. In pre-BOS (*P* = 0.98), BOS (*P* = 0.20), pre-RAS (*P* = 0.79), and RAS (*P* = 0.26), IL-1α was not different from the control; however, it was higher in BOS compared with pre-BOS (*P* = 0.015).

IL-1β levels were higher in LB/ARAD [23.11 pg/ml (0.53–269.11 pg/ml); *P* = 0.03] and PAN [63.11 pg/ml (11.21–1180.65 pg/ml); *P* < 0.0001] compared with the control [1.23 pg/ml (0.39–30.88 pg/ml)]. IL-1β was not different comparing the pre-BOS (*P* = 0.91), BOS (*P* = 0.22), pre-RAS (*P* = 0.48), and RAS (*P* = 0.26) group with the control group. IL-6 was increased in PAN [18.88 pg/ml (0–18.71 pg/ml); *P* = 0.0007] and RAS [6.820 pg/ml (0–371.1 pg/ml); *P* = 0.016] versus the control [1.14 pg/ml (0–8.4 pg/ml)]. IL-8 was increased in LB/ARAD [280.5 pg/ml (10.12–26887.19 pg/ml); *P* = 0.016], PAN [1073 pg/ml (325.22–22536.34 pg/ml); *P* < 0.0001], and BOS [47.84 pg/ml (9.21–499.1 pg/ml); *P* = 0.047] compared with control [11.95 pg/ml (3.33–337.31 pg/ml)]. TNF-α was only increased in PAN [24.13 pg/ml (9.89–159.35 pg/ml); *P* = 0.0002] compared with the control [1.78 pg/ml (0.46–22.11 pg/ml)] (Fig.3).

### Interaction of BAL with primary human bronchial epithelial cells

To investigate the interactions of BAL with primary human bronchial epithelial cells, PBEC were treated for 24 h with 1:1 BAL/media, after which cell viability was assessed using XTT toxicity assay. BAL samples were centrifuged, and only the supernatants were used in the PBEC stimulation. Epithelial cell viability was significantly different across the groups by Kruskal–Wallis anova (*P* = 0.034). The viability index of epithelial cells exposed to BAL was significantly lower in PAN [0.318 (0.162–0.457); *P* = 0.0094] compared with the control [0.416 (0.333–0.470)]. The viability index did not change for LB/ARAD (*P* = 0.64), pre-BOS (*P* = 0.52), BOS (*P* = 0.11), pre-RAS (*P* = 0.20), and RAS (*P* = 0.16) versus the control. The cell viability data are also presented as percentage change in OD compared with untreated controls (cell incubated in 1:1 media/PBS) (Fig. S1).

**Figure 4:**
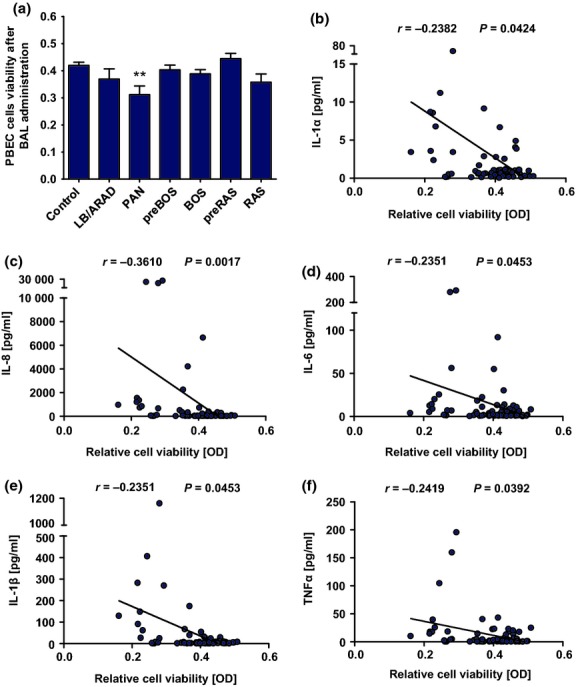
Influence of BAL from lung transplant patients on primary human bronchial epithelial cell (PBEC) viability. PBEC were cultured in 1:1 BAL/SABM media for 24 h at 37 °C, after which the cell viability was assessed with an XTT toxicity assay. Relative PBEC cell viability was expressed as optical density (OD) (a). Correlation between PBEC cell viability and IL-1α (b), IL-8 (c), IL-6 (d), IL-1β (e), and TNF-α (f) concentrations in BAL was assessed using Spearman's rank correlation coefficient. The different stars refer to the level of statistical significance (*P < 0.05, **P < 0.01, ***P <* *0.001).

**Figure 5:**
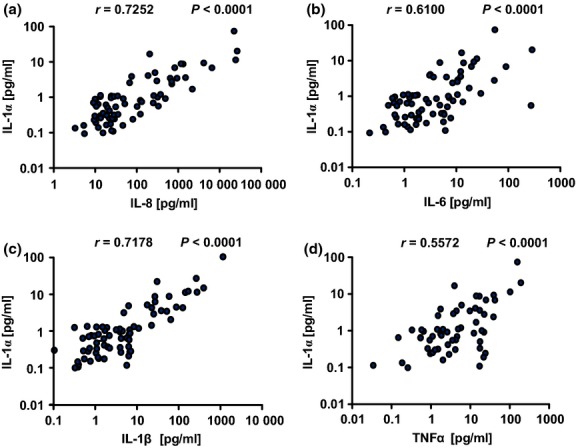
Correlation between a marker of epithelial injury, IL-1α, and proinflammatory cytokines in BAL of lung transplant patients. Correlation between IL-1α and IL-8 (a), IL-6 (b), IL-1β (c), and TNF-α (d) concentrations in BAL was assessed using Spearman's rank correlation coefficient. The analysis was performed in all groups together. The different stars refer to the level of statistical significance (*P < 0.05, **P < 0.01, ***P < 0.001).

Additionally, a spearman correlation between PBEC viability and concentrations of IL-1α, IL-1β, IL-6, IL-8, and TNF-α was performed (Fig.4b–f) in all groups together. The analysis revealed an inverse correlation between all the measured cytokines and PBEC viability (IL-1α, *r* = −0.24, *P* = 0.042; IL-8, *r* = −0.36, *P* = 0.0017; IL-6, *r* = −0.24, *P* = 0.045; IL-1β, *r* = −0.24, *P* = 0.045; and TNF-α, *r* = −0.24, *P* = 0.039). Previous studies revealed that epithelial damage is associated with release of alarmins including IL-1α 21. Therefore, we assessed the possible relationship between levels of the epithelial alarmin IL-1α and proinflammatory BAL cytokines. The analysis revealed a positive correlation between IL-1α and the measured cytokines (IL-8, *r* = 0.73, *P* < 0.0001; IL-6, *r* = 0.61, *P* < 0.0001; IL-1β, *r* = 0.72, *P* < 0.0001; and TNF-α, *r* = 0.56, *P* < 0.0001) (Fig.5).

## Discussion

Phenotyping acute and chronic rejection after lung transplantation has recently shown an important difference between A grade and B grade (or LB) acute rejection and between the distinct phenotypes of BOS, ARAD, and RAS 25–27. Lymphocytic bronchiolitis has been associated with chronic lung allograft rejection for more than 15 years 30. After the initial observation that airway neutrophilia predicts azithromycin responsiveness 25, we identified lung transplant recipients with LB and persistently increased airway neutrophilia, despite receiving azithromycin therapy and termed this new phenotype as persistent airway neutrophilia (PAN). Within BOS and RAS, we did not observe changes in alarmins or cytokines. Both had an increased BAL neutrophilia, and plasma CRP was also increased in RAS. Importantly, RAS also demonstrated an increased airway eosinophilia, which corroborates recent findings by our group 28. On the other hand, in the LB/ARAD and especially in PAN, upregulation of proinflammatory cytokines was present.

Recently, Verleden *et al*. 28 demonstrated that azithromycin treatment results in the reduction in IL-17-positive cells in the lung biopsies of lung transplant recipients with lymphocytic bronchiolitis/ARAD and not in PAN. Activation of IL-17 T cells and stimulation of IL-17 expression in neutrophils may be triggered by some intracellular bacteria 31 and viruses 32, which may be difficult to detect using standard microbiological methods. On the other hand, the presence of IL-17 T cells was not associated with infection in the aforementioned study. Previous and our recent observations suggest that chronic inflammatory lung diseases are associated with damage and disruption of the integrity of the respiratory epithelium and a release of epithelial alarmins including IL-1α. Chronic inflammation is also a hallmark of BOS; however, the levels of alarmins, proinflammatory cytokines, and immune cells in BALs of patients with different rejection phenotypes have not been investigated before. Therefore, the objective of this study was to assess IL-1α, proinflammatory cytokines, and immune cells in BALs of lung transplant recipients diagnosed with LB/ARAD, PAN, BOS, and RAS and to investigate the interactions between BAL from these different phenotypes and primary human bronchial cells.

Our study revealed that PAN is characterized by elevated levels of IL-1α, IL-1β, IL-8, IL-6, and TNF-α, of which the latter two seem only to be elevated in PAN. Moreover, PAN was the only phenotype in which the lavage fluid (containing airway surface liquid from airways as well as the alveolar space) caused decreased epithelial cell viability.

Both LB/ARAD and PAN are associated with increased numbers of BAL neutrophils. The main neutrophil chemoattractant, IL-8, was also significantly increased in BAL of these patients.

IL-8 is one of the most potent neutrophil chemoattractants, whose expression may be triggered by a variety of factors including IL-1α 33, TNF-α 34 and IL-1β 35. Additionally, previous reports demonstrated that there is a link between epithelial damage, inflammation, and neutrophilia 36. These reports are in agreement with our observations that there is an inverse correlation between PBEC viability and concentrations of proinflammatory cytokines in BALs. Identification of BAL-originated factors that trigger the epithelial damage would shed more light on the underlying cause of chronic inflammation and degeneration of the respiratory epithelium in patients with high neutrophilia who do not respond to azithromycin treatment.

Both IL-1α and IL-1β were significantly increased in PAN and LB/ARAD. The potential sources of IL-1α in PAN may be damaged epithelial cells. IL-1β and TNF-α may also originate from activated macrophages, and IL-1α may be released by macrophages and neutrophils undergoing apoptosis. Interestingly, TNF-α, which also may trigger IL-8 expression and contribute to neutrophil influx, was significantly increased only in PAN but not in LB/ARAD, suggesting that the pathogenesis of these two CLAD phenotypes may have different backgrounds; however, further studies are required to identify the mechanistic differences between these two phenotypes. Additionally, IL-8-mediated neutrophilia in LB/ARAD may be triggered by IL-17 secreted by Th17 cells whose presence was detected in lung sections from CLAD patients responsive to azithromycin 28.

Previous studies demonstrated that IL-17 expressing cells are present in the airways of LB/ARAD patients and that IL-17 induces expression of a neutrophil chemokine IL-8. It was also confirmed that interactions between neutrophils and T cells may modulate T-cell responses. Thewissen *et al*. 37 have shown that the presence of PMN in the co-culture dose-dependently increased the fraction of IFNγ and IL-17 producing T cells and decreased the percentage of IL-10 producing CD4(+) T cells, suggesting that persistent neutrophilia may polarize T cells into Th1/Th17 phenotype. Additionally, IL-1β that may be released by activated neutrophils may trigger expression of IL-17 in T cells and is required for Th17 cell differentiation and maturation. Moreover, IFNγ produced by Th1 and Th17 cells is required for the activation of M1 macrophages, which are a source of the neutrophil chemoattractant, IL-8 37.

Interestingly, our study demonstrated that proinflammatory cytokines did not change in BOS and RAS despite the presence of a neutrophilia.

In addition, the detected eosinophilia in RAS is a potential new concept to consider. Eosinophilia has been reported in the past in chronic rejection 38, yet never explored further. Classical BOS is becoming an increasingly difficult phenotype to increase mechanistic understanding of. Although more and more of the previously identified risk factors can be linked with the other phenotypes, ARAD (neutrophilia, IL-1β, IL-8, IL-6, and TNF-α, CRP) and RAS (eosinophilia, CRP), protein profiles observed in this and previous reports 27 do not show significant changes.

Considering that chronic lung rejection or CLAD may evolve to RAS or BOS, which are characterized by distinct histological features, it may be that genetic or epigenetic features determine the fate of the allograft after transplantation; however, further studies are required to test this hypothesis.

One of the limitations of the study was a small number of samples in each group. The groups were not significantly different except for age, which is an example of this small sample size effect. This manuscript does provide provocative data that should be validated in a larger multicenter study as it clearly demonstrates that different pathways are involved in LB/ARAD, PAN, BOS, and RAS, which may allow the development of targeted therapies in the future.

## Authorship

MIS and BMV: designed and performed the study, collected and analyzed the data, and wrote the article. SEV, RV, DEVR and GMV: designed the study and collected and analyzed the data. NJG: analyzed the data and wrote the article. CW: analyzed the data, PAC, AL, EV and LAB: contributed to the study design and editing the article. AJF: designed the study and wrote the article.

## Funding

Grants: GMV is holder of the GSK (Belgium) chair in respiratory pharmacology at the KULeuven and is supported by the university (OT OT10/050) and National Research Foundation Flanders (FWO): G.0723.10, G.0679.12, and G.0705.12. BMV is a senior research fellow and holder of a long-term traveling grant of the FWO. AJF is supported by Biotechnology and Biological Sciences Research Council. RV is supported by the Research Foundation Flanders (FWO) (KAN2014 1.5.139.14) and Klinisch Onderzoeksfonds (KOF) KULeuven. SEV is supported by the Onderzoeksfonds KULeuven.

## Supporting Information

Figure S1 PBEC were cultured in 1:1 BAL/SABM media for 24 h at 37°C, after which the cell viability was assessed with an XTT toxicity assay. The cell viability is presented as a percentage change in OD compared to untreated controls (cell incubated in 1:1 media/PBS).
