# Supplementary figures and images for: Mechanistic differences between phenotypes of chronic lung allograft dysfunction after lung transplantation
Source: Transpl Int. 2014 Jun 17;27(8):857–67. doi: 10.1111/tri.12341 (PMC4282071; doi:10.1111/tri.12341)

Supplemental figure1

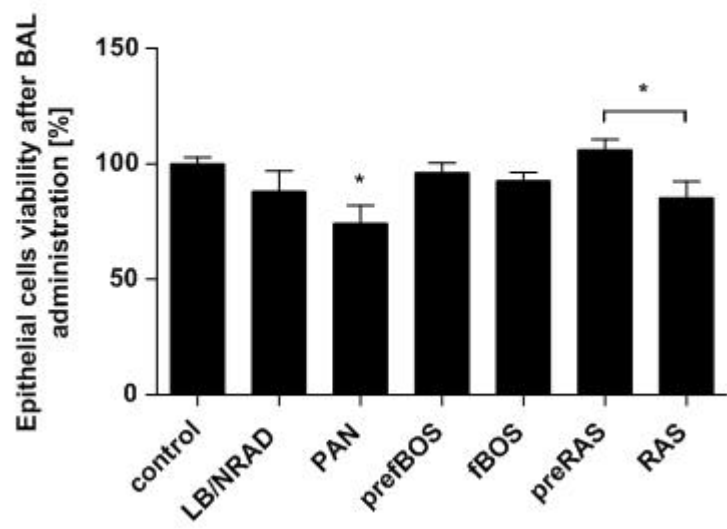

Supplement: Figure S1 — PBEC were cultured in 1:1 BAL/SABM media for 24 h at 37°C, after which the cell viability was assessed with an XTT toxicity assay. The cell viability is presented as a percentage change in OD compared to untreated controls (cell incubated in 1:1 media/PBS). [file tri0027-0857-SD1.pdf]
